# Electromagnetic Field Redistribution in Metal Nanoparticle on Graphene

**DOI:** 10.1186/s11671-018-2535-0

**Published:** 2018-04-25

**Authors:** Keke Li, Anping Liu, Dapeng Wei, Keke Yu, Xiaonan Sun, Sheng Yan, Yingzhou Huang

**Affiliations:** 1 Department of Applied Physics, College of Physics, Chongqing University, Chongqing, 400044 China; 2 Chongqing Key Laboratory of Multi-scale Manufacturing Technology, Chongqing Institute of Green and Intelligent Technology, Chinese Academy of Sciences, Chongqing, 400714 China; 3 Soft Matter and Interdisciplinary Research Center, College of Physics, Chongqing University, Chongqing, 400044 China; 4 Department of Physics, The Hong Kong University of Science and Technology, Clear Water Bay, Kowloon, Hong Kong China

**Keywords:** EM redistribution, Graphene plasmons, Nanoparticle/graphene hybrid system, SERS

## Abstract

Benefiting from the induced image charge on metal film, the light energy is confined on a film surface under metal nanoparticle dimer, which is called electromagnetic field redistribution. In this work, electromagnetic field distribution of metal nanoparticle monomer or dimer on graphene is investigated through finite-difference time-domain method. The results point out that the electromagnetic field (EM) redistribution occurs in this nanoparticle/graphene hybrid system at infrared region where light energy could also be confined on a monolayer graphene surface. Surface charge distribution was analyzed using finite element analysis, and surface-enhanced Raman spectrum (SERS) was utilized to verify this phenomenon. Furthermore, the data about dielectric nanoparticle on monolayer graphene demonstrate this EM redistribution is attributed to strong coupling between light-excited surface charge on monolayer graphene and graphene plasmon-induced image charge on dielectric nanoparticle surface. Our work extends the knowledge of monolayer graphene plasmon, which has a wide range of applications in monolayer graphene-related film.

**Electronic supplementary material:**

The online version of this article (10.1186/s11671-018-2535-0) contains supplementary material, which is available to authorized users.

## Background

As a pioneer of two-dimensional (2D) material in the twenty-first century, graphene possesses plenty of excellent properties, such as electronic performance, superior thermal conductivity, robust mechanical strength, and high surface area. Based on the aforementioned characteristics, graphene has attracted great interests and has been applied in various research fields, such as optoelectronics [1, 2], optical detection [3], solar cell [4–7], and energy storage [8, 9]. For the excellent ability of manipulating light in subwavelength, plasmonics that is based on surface plasmon induced by light at metal surface is also receiving considerable attention and has numerous applications, e.g. surface enhanced Raman scattering (SERS) [10, 11], sensor [12], surface catalysis [13], waveguide [14], and optical activity [15]. In recent years, the hybrid system consisting of graphene and metal nanoparticle has been studied extensively [16–21]. However, due to the effective coupling between metal particles, light energy is usually confined to the metal nanoparticle rather than on the graphene surface in nanoparticle/graphene hybrid system. In the last several years, a large amount of reports demonstrate that the inducing image charges on metal film generate light confinement on the metal film surface rather than on the nanoparticle in the nanoparticle/metal film hybrid system, which is called electromagnetic field redistribution [22–27]. Moreover, the result has also been demonstrated that nanoparticle dimer could represent the stronger light confinement phenomenon than nanoparticle monomer. In our work, nanoparticle/graphene hybrid systems are adopted to study the distribution of electromagnetic field on graphene. Firstly, the electric field and surface charge distributions in nanoparticle/graphene hybrid system were stimulated under different wavelength conditions using finite-difference time-domain (FDTD) method. And then, the phenomenon is further experimentally confirmed by the measured SERS. Furthermore, the results of the system of the dielectric dimer and graphene film point out the graphene plasmon, which induced image charge on nanoparticle, is crucial to this confinement. Our results in the work demonstrate the light energy can be focused on the monolayer graphene surface by Ag nanoparticle dimer at the infrared region both theoretically and experimentally, which has significant applications in monolayer graphene-related field.

## Methods/Experimental

### Material and Sample Preparation

AgNO_3_, polyvinylpyrrolidone (PVP), and sodium borohydride were purchased from Aldrich Chemical Co. The way to synthesize silver nanoparticles was through sodium borohydride reduction of AgNO_3._ The monolayer graphene was grown on clean copper foils in a tube-style chemical vapor deposition (CVD) system. Under atmospheric pressure, mixture gas of 25% methane in hydrogen (total flow rate of 80 sccm) was introduced into the chamber, after substrate temperature increased up to 1000 °C. The graphene growth maintained for 10–30 min, and then, the samples were cooled to room temperature rapidly. Finally, the graphene film was transferred onto the silicon substrate using poly(methyl-methacrylate) (PMMA) polymer [28]. Then, the nanoparticles are coated on single-layer graphene to form the nanoparticle-graphene hybrid structure.

### Simulation Parameter

In this work, all theoretical analyses were simulated adopting the FDTD method to calculate the distributions of electric field and the surface charge. The Ag nanoparticle dimer was located 1 nm above the graphene, and the gap of Ag nanoparticle dimer was also set as 1 nm. The PVP on the particles is 0.5 nm, and the spacing of the graphene is 0.5 nm. Thus, we set the gap as 1 nm. The Si is underneath the monolayer. Then, the 633, 2000, and 3000 nm plane light source perpendicularly irradiated to the substrate, respectively. The electric component (*E*_0_) of plane source was set as 1 V/m. Then, the conditions of FDTD boundary were set to perfectly matched layer (PML), which absorbed total incident light. Moreover, the frequency-domain field profile monitors were added to superintend the electromagnetic field distributions for succedent analysis. The excited light entered from the Ag nanoparticle side normal to the substrate plane. The polarization of incident light is along the dimer axis, which can effectively excite the plasmon coupling modes in the dimers.

## Results and Discussion

**Fig. 1:**
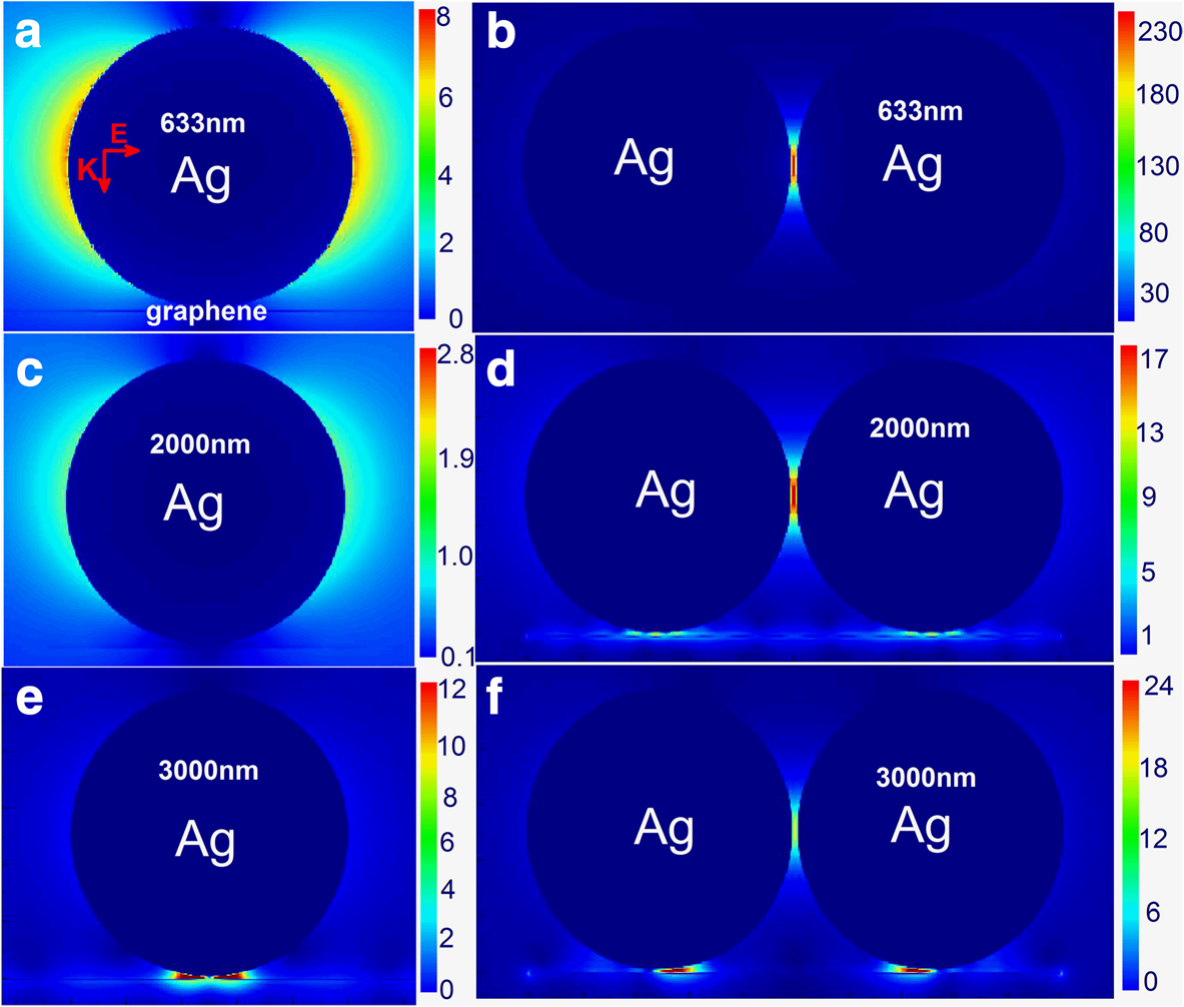
Electric field redistribution in nanoparticle/graphene hybrid system. **a**, **b** Electric field distributions of *R* = 50 nm nanoparticle monomer and dimer on monolayer graphene film with a 1-nm gap at 633 nm, **c**, **d** at 2000 nm, and **e**, **f** at 3000 nm

As shown in Fig. 1, the electric field distributions of Ag nanoparticle monomer and dimer on monolayer graphene film hybrid systems were calculated under the excitation wavelengths of 633, 2000, and 3000 nm, separately. Figure 1a shows the electric field distribution of the system, which includes the 100-nm Ag nanoparticle monomer on monolayer graphene film at 633 nm. The distribution of the electric field totally localizes at the sides of the nanoparticle but hardly exists in the gaps of particle film. The electric field distribution on the Ag nanoparticle dimer at 633 nm is shown in Fig. 1b. The electric field is mainly confined to the gap of the particle-particle. Comparing with the electric field of the gap of the particle-particle, the electric field outside the gap of the particle-particle could be extraordinarily weak. And in Fig. 1b, the one scale bar was used to describe the electric field intensity of the gaps of particle-particle and particle-graphene. Thus, the obvious electric field enhancement could not be seen. At 633 nm, the electric field enhancement merely comes from effective coupling of particle-particle rather than coupling of particle-film in the system, so the light energy is primarily confined in the gap of particle-particle. When the wavelength is changed to infrared wavelength, 2000 nm, the distributions of electric field are exhibited in Fig. 1c, d. The wavelength change leads to the electric field redistribution of the system. In Fig. 1d, the dimer on the monolayer graphene produces more pronounced effect than only using monomer case. The distribution of the electric field not only exists on the sides of the particle, but also in the gap of the particle-film. Although electric field enhancement of the gap of particle-film is weaker than the one in the gap of particle-particle, it cannot be ignored. The result proves that monolayer graphene plasmons have produced the effective effect on the electric field enhancement of the system and the light energy is confined to the gap of particle-particle and the graphene surface at 2000 nm. Afterward, the infrared light source, 3000 nm wavelength, is used and results are shown in Fig. 1e, f. Figure 1e, f describes that the strongest electric field enhancement generates in the gap of particle-film at 3000 nm. Therefore, the light energy is confined in the monolayer graphene surface at 3000 nm. The electric field distributions in the case of more wavelengths are placed in Additional file 1. Compared with electric field distributions under different wavelength conditions, it is found that the light energy is better focused on the monolayer graphene at 3000 nm. Furthermore, because the 633 nm is closer to the resonate peak of Ag nanoparticle, the electric field enhancement factor is 2.3 × 10^2^ at 633 nm, which is stronger than that excited at 3000 nm. The simulation results reveal the feature of the system: changing the laser wavelength could lead to electric field redistribution, which avails the light energy focusing on the graphene surface. The reason for generating the phenomenon is due to the permittivity of the monolayer graphene under different wavelength conditions. At 633 nm, the permittivity of the monolayer graphene is 1.539, which expresses the property of dielectric. However, the monolayer graphene permittivity is − 19.083 at 3000 nm, which is similar to metal. The property of monolayer graphene under different wavelengths leads to the electric field redistribution of the system. The previous studies demonstrate the effective coupling of particle and film plays an important role in this system comprising of a gold film with a 100-nm Ag nanoparticle dimer located 1 nm above the film at 633 nm. Therefore, the light energy could be focused on the gold film under the metal nanoparticle dimer [28]. In comparison of aforementioned consequences, it is evident that the light energy could mainly be focused on the monolayer graphene surface by the metal dimer at the infrared region.

**Fig. 2:**
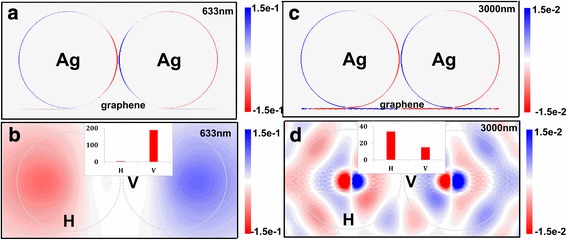
Surface charge redistribution in nanoparticle/graphene hybrid system. Surface charge distributions of *R* = 50 nm Ag nanoparticle dimer on monolayer graphene with 1-nm gap **a** at 633 nm and **c** at 3000 nm. Surface charge distributions in graphene surface of *R* = 50 nm Ag nanoparticle dimer on monolayer graphene with 1-nm gap **b** at 633 nm and **d** at 3000 nm. The deduced charge dipole interaction representation at 633 and 3000 nm is shown on the right of Fig. 2

To deeply understand the physical mechanism of above phenomenon, surface charge distributions of dimer-film system at various wavelengths were stimulated in Fig. 2. As shown in Fig. 2a, a large amount of free electrons are confined to the surface of the nanoparticle. However, with the change of excited wavelength, most of free electrons gather on the monolayer graphene surface at 3000 nm in Fig. 2c, and the surface charge distribution of nanoparticle at 633 nm presents stronger localization effects than that at 3000 nm. The results are further confirmed in Fig. 2b, d, which describes the surface charge distribution of the monolayer graphene at 633 and 3000 nm in the 100-nm Ag nanoparticle dimer/gold film hybrid system, respectively. At 3000 nm, the free electrons of the system primarily gather at the bottom of the nanoparticles to form a relatively strong coupling with the monolayer graphene, which leads the electric field enhancement of system mostly localized at the gap of particle-film. Then, the scale bar of the surface charge distribution of the system and the scale bar of surface charge distribution of the monolayer graphene are uniform under the same excited wavelength conditions. In the comparison of Fig. 2b, d, it is not found that the proportion accounting for the system of the charges gathering on monolayer graphene surface at 633 nm is less than the proportion at 3000 nm. The insets in Fig. 2b, d present the electric field intensity of the horizontal and vertical gap at 633 and 3000 nm, respectively. At 633 nm, the electric field enhancement of the vertical gap is stronger than that of the horizontal gap, which demonstrates that the light energy is focused on the horizontal gap. All in all, at visible range, the dipole hybridization of particle-particle makes free electrons gather at the nanoparticles, which leads to the strong electric field enhancement in the gap of particle-particle in the Ag nanoparticle dimer/graphene hybrid system. At the infrared region, because the metal property of graphene and the Ag nanoparticle is far from the resonant peak, free electrons on the graphene surface induce image charges on the nanoparticle surface. Thus, coupling of free electrons on the graphene surface and image charges on the nanoparticle surface generates the electric field enhancement in the gaps of particle-film. The results also demonstrate the light energy could be confined on the graphene surface at the infrared region.

**Fig. 3:**
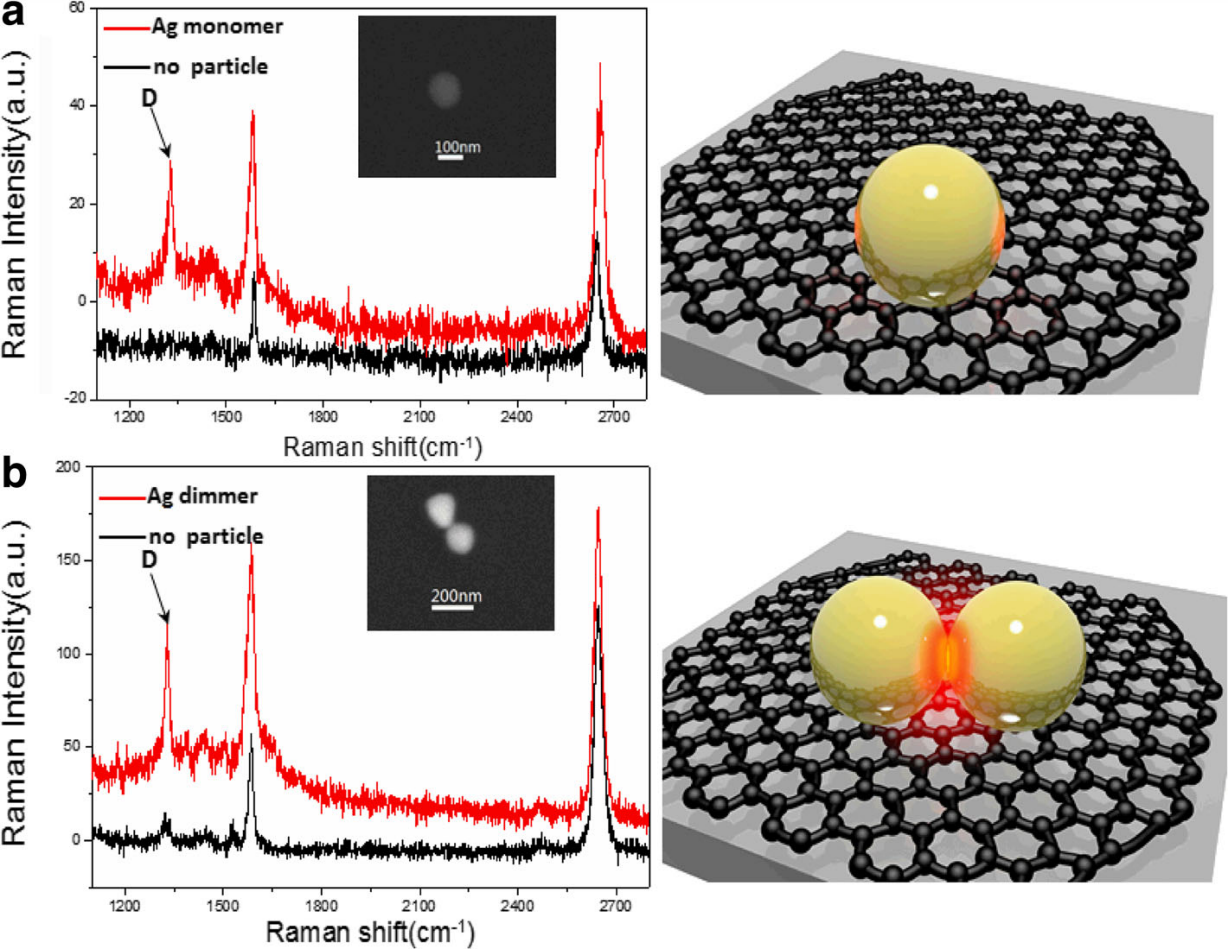
SERS of nanoparticle/graphene hybrid system. **a** SERS of monolayer graphene adsorbed on graphene from Ag nanoparticle monomer and without particle and scheme of the samples. **b** SERS of monolayer graphene adsorbed on graphene from Ag nanoparticle dimer and without particle and scheme of the samples

In Fig. 3, the scale bars in SEM (scanning electron microscope) images point out the Ag nanoparticle monomer and dimer have similar diameter about 100 nm. The SERS spectra in Fig. 3a come from the regions where Ag nanoparticle monomer and without particle are on monolayer graphene, respectively. The purpose we also collect the Raman of graphene without Ag particle is to highlight that Ag nanoparticle could enhance the Raman signal through the electric field enhancement. The schematic diagram of the system is displayed on the right of Fig. 3a. The stronger Raman intensity illustrates that Ag nanoparticle monomer can generate Raman enhancement. To further verify the result, the Raman spectra of monolayer graphene with Ag nanoparticle dimer are also measured in Fig. 3b. The schematic diagram of the system is similarly displayed on the right of Fig. 3b. The obvious Raman enhancement effect is also observed in Fig. 3b, which is consistent with Fig. 3a. These results also demonstrate the Raman signal of monolayer graphene can be improved by Ag nanoparticles. But there is a discrepancy that the simulating enhancement factor of dimer in Fig. 1b is larger than the experimentally determined one in Fig. 3b. On the one hand, the electric field enhancement is localized in the gap of particle-particle in Fig. 1b, but the nanoparticles are deposited on the surface of graphene in the experiment. Thus, the discrepancy mainly comes from the difference of region of electric enhancement field and contact surface. The result verifies that the gaps of particle-film do not generate electric field enhancement and further demonstrates light energy is confined in the gap of particle-particle at 633 nm. On the other hand, the ideal geometric parameters of the nanostructures are used in the simulations, though it is hard to achieve in the actual experiments. Moreover, the shape, surface roughness, and the gap of particle-particle could also affect the enhancement factors, which may cause the unconformity. It is worth noting that the D band of monolayer graphene was largely induced when Raman spectroscopy of Ag nanoparticle-graphene was measured. The proper theoretical explanation of the phenomenon is free electrons of Ag nanoparticles can lead to stronger energy to effectively arouse D band of monolayer graphene.

**Fig 4:**
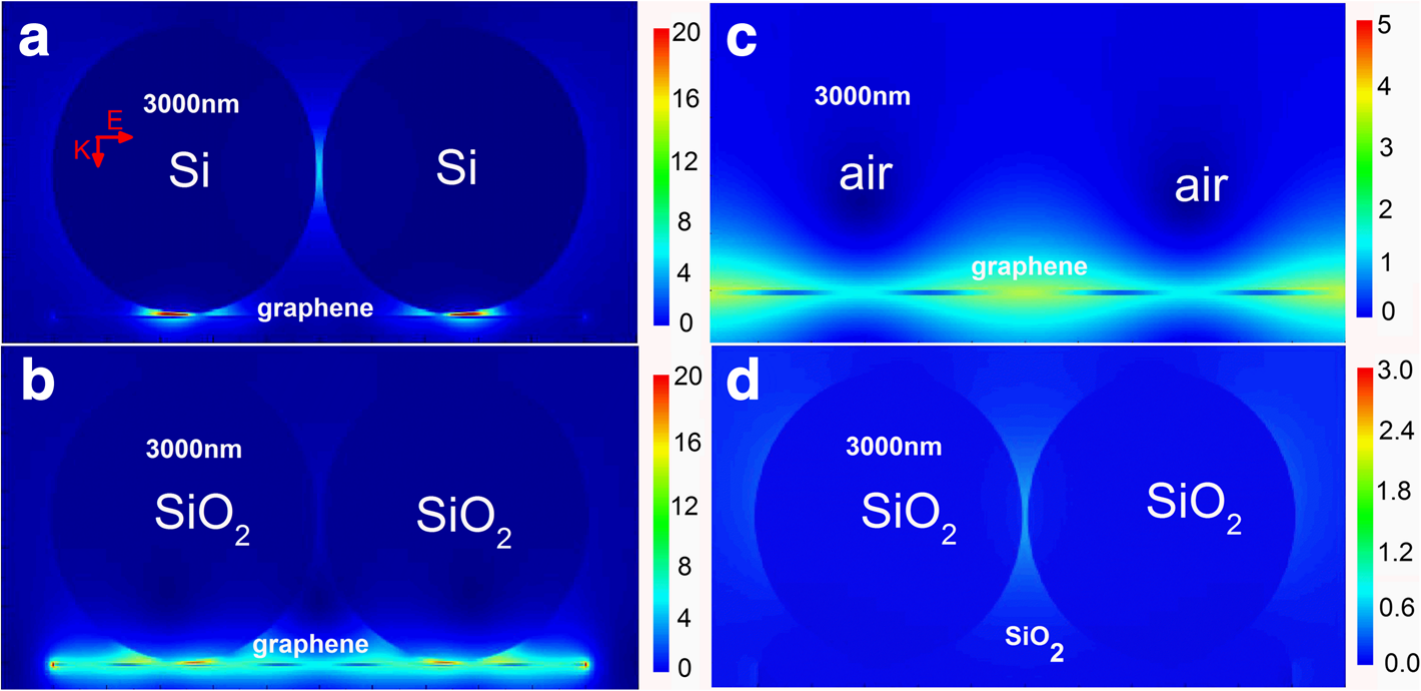
Electric field distributions in different dielectric nanoparticle/graphene hybrid system. **a**–**c** Electric field distributions of different permittivity nanoparticle dimers on monolayer graphene film with a 1-nm gap at 3000 nm. **a** Si (*n* = 4.21 + 0.017i), **b** SiO_2_ (*n* = 1.5), **c** air (*n* = 1). **d** Electric field distributions of SiO_2_ nanoparticle dimer on SiO_2_ film with a 1-nm gap at 3000 nm

Aforementioned results reveal that free electrons on graphene surface induce image charges on nanoparticle surface at the infrared region and the result is further confirmed in Fig. 4. Figure 4a, b describes the electric field distributions of the system at 3000 nm, which are comprised of different permittivity nanoparticle dimers and monolayer graphene film. As shown in Fig. 4a, b, with the permittivity of the nanoparticle dimer decreasing, the electric field enhancement of system will also become very weak. In Fig. 4c, the electric field enhancement of monolayer graphene without nanoparticle was also simulated, which is weaker than that in Fig. 4a, b. Then, Fig. 4d shows the electric field distribution of the system, which is comprised of SiO_2_ nanoparticle dimer on the SiO_2_ film. The dimer and film are both nonconductors, which hardly generate electric field enhancement. The gaps of particle-particle and particle-film in the systems were both set as 1 nm. In comparison to Fig. 4b, d, the stronger electric field enhancement in Fig. 4b illustrates that light energy is confined on monolayer graphene film only when using the monolayer graphene as film at 3000 nm. The aforementioned result demonstrates the graphene plasmons could be induced at infrared region, which can produce effective coupling with image charges in the nanoparticle dimer. However, in Fig. 4c, the system does not exist nanoparticle dimer, which leads that the monolayer graphene plasmons cannot induce image charges. The phenomenon further verifies that light energy could be confined on the monolayer graphene film at infrared region in this nanoparticle/graphene hybrid system. Furthermore, in Fig. 4a, b, the Si nanoparticle dimer as semiconductor produces more image charges than SiO_2_, so the electric field enhancement of Si nanoparticle/graphene hybrid system is stronger and shows the better localization effect in the gap of particle-film. These results possess profound meanings to the application of monolayer graphene.

## Conclusion

In summary, the electromagnetic field distribution of the hybrid system between graphene and metal nanoparticles has been investigated in this work. The results indicate the light confined by surface plasmon is influenced by the wavelength. That is, light confinement is at the gap between nanoparticles at visible region and at graphene surface at infrared region. Our work extends the knowledge of graphene plasmon, which has wide application aspects in graphene-related film.

## Additional File

Additional file 1: **Figure S1.** a Electric field distributions of *R* = 50 nm nanoparticle dimer on monolayer graphene film with a 1-nm gap at 785 nm, b at 1080 nm, c at 1580 nm, and d at 5000 nm. **Figure S2.** Electric field distributions of *R* = 50 nm nanoparticle monomer on SiO_2_ film with a 1-nm gap at 2000 nm. **Figure S3.** SERS of monolayer graphene adsorbed on graphene from Ag nanoparticle monomer and without particle and scheme of the samples. (DOCX 302 kb)

## Abbreviations

CVD: Chemical vapor deposition
EM: Electromagnetic field
FDTD: Finite-difference time-domain
PML: Perfectly matched layer
PMMA: Poly(methyl-methacrylate)
SEM: Scanning electron microscope
SERS: Surface-enhanced Raman spectrum
