# Supplementary material for: Electromagnetic Field Redistribution in Metal Nanoparticle on Graphene
Source: Nanoscale Res Lett. 2018 Apr 25;13:124. doi: 10.1186/s11671-018-2535-0 (PMC5918144; doi:10.1186/s11671-018-2535-0)
Supplement: Supplementary file 1 — Figure S1. a Electric field distributions of R = 50 nm nanoparticle dimer on monolayer graphene film with a 1-nm gap at 785 nm, b at 1080 nm, c at 1580 nm, and d at 5000 nm. Figure S2. Electric field distributions of R = 50 nm nanoparticle monomer on SiO2 film with a 1-nm gap at 2000 nm. Figure S3. SERS of monolayer graphene adsorbed on graphene from Ag nanoparticle monomer and without particle and scheme of the samples. (DOCX 302 kb) [file 11671_2018_2535_MOESM1_ESM.docx]

**Additional file 1**

**for**

**Electromagnetic Field Redistribution in Metal Nanoparticle on Graphene**

Keke Li^1,3^, Anping Liu^1^*, Dapeng Wei^2^*, Keke Yu^1,3^, Xiaonan Sun^1^, Sheng Yan^4^ and Yingzhou Huang^3^*

*^1^ Applied of Physics, College of Physics, Chongqing University, Chongqing, 400044, China*

*^2^ Chongqing Key Laboratory of Multi-scale Manufacturing Technology, Chongqing Institute of Green and Intelligent Technology, Chinese Academy of Sciences, Chongqing 400714, PR China*

*^3^ Soft Matter and Interdisciplinary Research Center, College of Physics, Chongqing University, Chongqing, 400044, China*

*^4^Department of Physics, The Hong Kong University of Science and Technology, Clear Water Bay, Kowloon, Hong Kong, China*

**Electromagnetic Field Redistribution in Metal Nanoparticle on Graphene**

Figure S1 exhibits the electric field distributions of Ag nanoparticle dimer on monolayer graphene film hybrid systems under the other excitation wavelengths conditions except for 633, 2000 and 3000nm. At 785 and 1080 nm, the electric field is mainly confined in the gap of the particle-particle, which is similar to the electric field distribution at 633nm. However, compared with the factor of electric enhancement at 633nm, the factors of electric field enhancement at 785 and 1080nm are weak. Then, at 1580nm, the electric field enhancement begins to exist in the gap of the particle-film. However, at 5000nm, the distributions of electric filed exist on the sides of the particle and the in the gap of the particle-film, which is similar to the electric field distribution at 2000 nm.





Figure S1. (a) Electric field distributions of R=50 nm nanoparticle dimer on monolayer graphene film with a 1 nm gap at 785 nm (b) at 1080 nm (c) at 1580nm and (d) at 5000 nm.

FigureS2 shows the electric field distribution of system, which includes the 100 nm Ag nanoparticle monomer on SiO_2_ film at 2000 nm. The distribution of the electric field mainly localizes at the sides of the nanoparticle and hardly exists in the gaps of particle-film.





**Figure S2.** Electric field distributions of R=50 nm nanoparticle monomer on SiO_2_ film with a 1 nm gap at 2000 nm.

In Figure S3, the SERS spectra obatined from monolayer graphene with Ag nanoparticle monomer and without Ag nanoparticle, respectively. It is evident that the presence of Ag nanoparticle monomer aroused D peak. Although the factor of the SERS is relatively small, the Ag nanoparticle monomer could still generate the effect of the SERS.

**

**

**Figure S3**. SERS of monolayer graphene adsorbed on graphene from Ag nanoparticle monomer and without particle and scheme of the samples.

Under different wavelength, the absorptivity of the Ag nanoparticle is different. At 633nm, the absorptivity of the Ag nanoparticle monomer is 0.55, and the absorptivity of the Ag nanoparticle dimer is 0.51. At 2000nm, the absorptivity of the Ag nanoparticle monomer is 0.9745, and the absorptivity of the Ag nanoparticle dimer is 0.9936. At 3000nm, the absorptivity of the Ag nanoparticle monomer is 0.9848, and the absorptivity of the Ag nanoparticle dimer is 1.
